# Effect of Polymer Dissolution Temperature and Conditioning Time on the Morphological and Physicochemical Characteristics of Poly(Vinylidene Fluoride) Membranes Prepared by Non-Solvent Induced Phase Separation

**DOI:** 10.3390/polym13234062

**Published:** 2021-11-23

**Authors:** João Teixeira, Vanessa Fernandes Cardoso, Gabriela Botelho, António Miguel Morão, João Nunes-Pereira, Senentxu Lanceros-Mendez

**Affiliations:** 1 CF-UM-UP, Centre of Physics of Minho and Porto Universities, Campus de Gualtar, University of Minho, 4710-057 Braga, Portugal; Id8042@alunos.uminho.pt (J.T.); vanessa@dei.uminho.pt (V.F.C.); 2 CMEMS-UMinho, Campus de Azurém, University of Minho, 4800-058 Guimarães, Portugal; 3 Department of Chemistry, Campus de Gualtar, University of Minho, 4710-057 Braga, Portugal; gbotelho@quimica.uminho.pt; 4 CICS-UBI, The Health Sciences Research Centre, University of Beira Interior, Av. Infante D. Henrique, 6200-506 Covilhã, Portugal; ammorao@yahoo.com.br; 5 C-MAST-UBI, Centre for Mechanical and Aerospace Science and Technologies, University of Beira Interior, Rua Marquês d’Ávila e Bolama, 6200-001 Covilhã, Portugal; 6 BCMaterials, Basque Center for Materials, Applications and Nanostructures, UPV/EHU Science Park, 48940 Leioa, Spain; 7 IKERBASQUE, Basque Foundation for Science, 48009 Bilbao, Spain

**Keywords:** poly(vinylidene fluoride), membranes, non-solvent induced phase separation, dissolution temperature, conditioning time

## Abstract

This work reports on the production of poly(vinylidene fluoride) (PVDF) membranes by non-solvent induced phase separation (NIPS) using *N*,*N*-dimethylformamide (DMF) as solvent and water as non-solvent. The influence of the processing conditions in the morphology, surface characteristics, structure, thermal and mechanical properties were evaluated for polymer dissolution temperatures between 25 and 150 °C and conditioning time between 0 and 10 min. Finger-like pore morphology was obtained for all membranes and increasing the polymer dissolution temperature led to an increase in the average pore size (≈0.9 and 2.1 µm), porosity (≈50 to 90%) and water contact angle (up to 80°), in turn decreasing the β PVDF content (≈67 to 20%) with the degree of crystallinity remaining approximately constant (≈56%). The conditioning time did not significantly affect the polymer properties studied. Thus, the control of NIPS parameters proved to be suitable for tailoring PVDF membrane properties.

## 1. Introduction

Membranes have received increasing attention since the development of the first large-scale commercial membranes in the mid 1960s, when they started to be used in micro- and ultrafiltration processes. Since then, these applications have become common in many applications and markets [1] and, in particular, polymer membrane technologies have expanded to practically every industrial sector including environmental, electronic or biotechnological ones [2]. Some of the most important membrane applications include batteries [3,4,5], fuel cells [6,7], filtration [8,9], air filtration [10,11], water treatment [12,13,14], agriculture [15,16], pharmacy [17,18], tissue engineering [19,20], biomedical applications [21,22], among others.

The selection of the material and the preparation process are key factors to develop membranes with suitable requirements for specific applications [23]. PVDF is a polymer of growing interest for membrane fabrication, based on its low surface energy, relatively high hydrophobicity, high mechanical strength, chemical resistance and thermal stability compared to other commercial polymer materials [2,24,25]. It also shows a very good processability as film [26,27], membranes [28,29,30], electrospun membranes [31,32], hollow fibres [33,34] or tubular membranes [35]. The non-solvent induced phase separation (NIPS) process is widely used to produce the membranes; in this process a polymeric solution contacts with a non-solvent inducing a phase separation into two different phases, one with high concentration of polymer and another with low concentration, enabling the formation of polymeric porous structures [2,36,37,38]. The NIPS process has been the subject of several studies, by itself or in parallel with TIPS (thermally induced phase separation) [39,40], a process with which NIPS commonly competes during membrane formation [40]. During the NIPS process there are several parameters that can influence the final morphology, surface and bulk properties of the membranes such as the polymer concentration, types of solvent, the coagulation bath medium and temperature, and non-solvent additives, among others [2].

Regarding the solvent and polymer interactions, relevant works have already been reported [41,42] broadly testing different solvent–polymer systems and identifying those that presents superior results in terms of solubility parameters, according to Hansen space. The aprotic solvent used in this work, *N*,*N*-dimethylformamide (DMF), ranks as one of the solvents with the highest total solubility parameter, 24.8 MPa^1/2^ [42]. The solvent is a key factor in determining the morphology and final properties of the membrane, it must easily dissolve or disperse the polymer and simultaneously should be miscible with the non-solvent [43]. Alternatively, a mixture of solvents have been used, as in the case of DMF and γ-butyrolactone (γ-BL) in which a relative concentration of 8/2 of DMF to γ-BL led to the highest tensile strength (7.3 MPa) and stress at break (223%) in the samples obtained [44]. Recently, efforts to make membrane manufacturing sustainable has leveraged the search for more ecofriendly solvents that can be viable alternatives in these fabrication processes [45], such as the Rhodiasolv PolarClean [46,47], Tamisolve NxG [28] and triethyl phosphate [48] denominated green solvents or non-toxic solvents that are less toxic than the conventional ones and present suitable properties for PVDF membrane preparation via the NIPS method.

The coagulation medium is decisive for the liquid–liquid demixing mechanism during the membrane formation in the NIPS process. The use of water as the non-solvent results in a rapid liquid–liquid demixing providing the formation of an asymmetric membrane [2]. The temperature of the coagulation bath influences decisively the final morphology of the membranes, with high temperature favoring the appearance of the asymmetric fingerlike morphology [49,50]. Temperature increasing from 20 to 60 °C leads to a decrease above 40% of β-PVDF content, an increase between 5 to 30° of the contact angle, and a decrease between 0.12 and 0.24 µm of the main pore size [51]. The increase of PVDF membrane porosity (>70%), tensile strength (6.21 MPa) and elongation at break (>74%) was reported as an effect of increasing temperature of the water coagulation bath up to 60 °C [52]. The enhancement of the mechanical properties is contrary to the general trend commonly reported in the literature. The coagulation bath medium has an influence in the phase inversion process of membrane formation. Water is a strong non-solvent that is extensively used in these processes, nonetheless the addition of different solvents in the water bath or the use of solvents in the coagulation bath have been studied [2]. The use of 1-octanol as coagulation bath medium is well reported in the literature, the use of this solvent leading to the formation of a symmetric cross-section PVDF membrane with spherulitic structure owing to the precipitation process governed by a crystallization mechanism [53,54]. A coagulation bath of ethanol, compared with water, leads to PVDF membranes with symmetrical cross-section structures and high hydrophobic surfaces (contact angle > 136°), with higher water flux and porosity [55]. The addition of ethanol 96% to deionized water lowers the solvent and non-solvent exchange rate, delaying the liquid-liquid demixing, which results in a markedly increase of the hydrophobicity from ≈84 to 150° contact angle [56]. Carbon nanosphere (CNS) sol was used in the coagulation bath allowing simultaneously the immobilization of the nanospheres into the PVDF membranes and the tailoring of physicochemical properties, increasing the hydrophilicity for small dosages of CNS (up to 400 mg/L) and decreasing porosity and permeability for higher dosages (800 mg/L) [57]. Despite the influence that all these parameters on the final properties of the membranes, not all of them have been studied in depth. The effect of the polymer temperature dissolution and the conditioning time before coagulation has been rarely reported, and specifically for the system DMF/PVDF/water, the studies are scarce, which makes this study necessary and relevant.

The study of the effect of these parameters on final membrane characteristics can be supported by the solution thermodynamics considering the interactions between solvent, polymer and non-solvent. The understanding of the final properties of the membranes can be improved with the ternary phase diagram of the system, since it can be used to predict the system’s interactions and the final properties of the membranes. In this context, there are relevant contributions in the literature for the system studied in this work and related ones [40,50,58,59].

The use of the NIPS process has increased significantly in the manufacture of polymer electrolyte membranes for energy storage systems such as batteries and full cells, among others. Thus, different polymer/solvent/non-solvent systems have been investigated. Some relevant studies include the binary composition of PVDF and N-methyl-2-pyrrolidone/acetone with the non-solvent mixture of ethanol/water, leading to improved porosity and electrolyte uptake for lithium-ion battery (LIB) [60]. Further, polyether block amide reinforced with bacterial cellulose nanocrystals dispersed in DMF and coagulated with deionized water have been investigated, leading to microporous membranes with superior wettability, ionic conductivity and thermal stability for LIB [61]. Polybenzimidazole porous membranes have been produced via a two-step NIPS process with the solvent *N*,*N*-dimethylacetamide and the non-solvents n-heptane, 1,2-dibromoethane and water, allowing membranes with improved ion selectivity and proton conductivity to be obtained for a vanadium flow battery [62]. Porous electrode structures based on polyacrylonitrile were prepared by dissolution in N-methyl-2-pyrrolidone with Cu powder added, followed by coagulation with non-solvent deionized water. The three-dimensional porous membranes show a bicontinuous microstructure and a suitable pore size and mechanical properties [63].

In this sense, this study reports the production of PVDF membranes via NIPS using DMF as solvent and water as non-solvent. In particular, the influence of dissolution temperature and conditioning time before the coagulation bath on the morphology, surface characteristics, structural, thermal and mechanical properties of the membranes have been addressed as relevant membrane parameters for a wide variety of applications.

## 2. Materials and Methods

### 2.1. Materials

PVDF 1010 polymer powder (352 kg/mol, ≥99.9%) were obtained from Solvay (Brussels, Belgium) and *N*,*N*-dimethylformamide (DMF, 99.5%) from Merck (Darmstadt, Germany). Ultrapure water was obtained through a water purification system from Millipore (Burlington, MA, USA). PVDF and DMF were used without any further treatments.

### 2.2. Membrane Preparation

**Figure 1:**
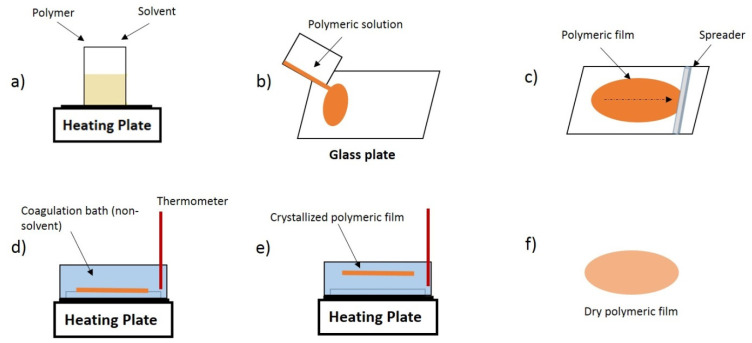
Schematic representation of the sample preparation steps by non-solvent induced phase separation (NIPS): (**a**) polymer dissolution, (**b**) solution spreading, (**c**) solution casting, (**d**) immersion in the coagulation bath, (**e**) phase inversion and (**f**) membrane drying.

PVDF membranes were prepared by NIPS [38] according to the following experimental procedure (Figure 1): PVDF was dissolved in DMF with a volume fraction (vol.%) of 9% under magnetic stirring (Ika C-Mag HS7, Staufen, Germany) at different temperatures (25, 50, 75, 100, 125 and 150 °C) and during a fixed time of 4 h to systematically evaluate the influence of dissolution temperature in the final membrane structure. The polymer solution was then deposited on a clean glass substrate and uniformly spread using a homemade casting knife with a 450 µm gap. The resultant assembly was immersed in a recipient filled with ultrapure water (non-solvent) at 75 °C to induce the phase separation process. The time between the end of the solution spreading on the glass substrate and the immersion in the coagulation bath was varied between 0 and 10 min to study the effect of this conditioning time on the membrane properties. During crystallization in the non-solvent, the membranes slowly split apart from the glass substrates. After that, the membranes were washed carefully with water to remove traces of solvents and finally air dried at room temperature for 24 h. Glass plates were used as substrates for being amorphous, and thus not contributing to the preferential crystallization of a particular phase of PVDF [64].

As previously stated, during sample preparation, the temperature of the polymer dissolution (between 25 °C and 150 °C) and the conditioning time on the glass plate before immersion (between 0 and 10 min) were varied, while the dissolution time was kept constant at 4 h. In this way, the effect of these parameters in the morphological, structural, thermal and mechanical properties of the membranes could be addressed. The coagulation bath was kept constant at 75 °C since at this temperature membranes with homogeneous and uniform surfaces are obtained, while at lower temperatures the membranes become wrinkled and irregular [64].

Next, the membranes were identified as a/b where a represents the dissolution temperature in °C and b the conditioning time in minutes between the spreading of the solution on the glass substrate and its immersion in the coagulation bath.

### 2.3. Membrane Characterization

Membrane morphology was evaluated using scanning electron microscopy (SEM). The images were obtained using a Thermo Fisher Scientific Quanta 650 FEG scanning electron microscope (Waltham, MA, USA) with 15 kV of acceleration voltage. Pore size was analysed using the ImageJ software (National Institutes of Health, Laboratory for Optical, Bethesda, MD, USA; and Computational Instrumentation, University of Wisconsin, Madison, WI, USA) with 40 measurements per image and the results are presented as the average and standard deviation.

To determine the PVDF polymer phase and content, Fourier transformed infrared spectroscopy (FTIR) measurements in the attenuated total reflection (ATR) mode were performed in a Jasco FT/IR-4100 (Pfungstadt, Germany) in the spectral range of 4000–600 cm^−1^ with 32 scans at a resolution of 4 cm^−1^. The spectra of the FTIR-ATR were used to determine the crystalline phase contents present in the PVDF membranes. Taking into consideration the 763 and 840 cm^−1^ absorption bands attributed to α and β phases, respectively, the amount of each phase can be calculated. Using the Lambert–Beer law and the absorption coefficients K_α_ and K_β_ (6.1 × 10^4^ cm^2^/mol and 7.7 × 10^4^ cm^2^/mol at 763 and 840 cm^−1^, respectively) the phase content is determined by Equation (1) [65]:

(1) $$F\left(\beta \right)=\frac{A_{\alpha }}{\left(\frac{K_{\beta }}{K_{\alpha }}\right)A_{\alpha }+A_{\beta }}\times 100,$$

where F(β) is the polar and electroactive β phase percentage, and A_α_ and A_β_ are the absorbances at 763 and 840 cm^−1^ respectively.

Differential scanning calorimetry (DSC) was used to evaluate the melting enthalpy and the degree of crystallinity of the samples. A sweep between 25 and 200 °C at a heating rate of 20 °C/min was performed using a Perkin-Elmer DSC 8000 (Waltham, MA, USA) under nitrogen flow, in perforated 30 µL heating pans to allow the release of volatile products. Through the obtained thermograms, the degree of crystallinity (χ_c_) was determined by Equation (2):

(2) $$\chi_{c}\left(\%\right)=\frac{\Delta H_{f}}{x\Delta H_{\alpha }+y\Delta H_{\beta }}\times 100,$$

where, according to FTIR-ATR measurements, x and y are the fraction of α and β phases of PVDF, respectively. $\Delta H_{f}$ is the melting enthalpy of the samples and $\Delta H_{\alpha }$ (93.04 J/g) and $\Delta H_{\beta }$ (103.4 J/g) are the melting enthalpies of α and β phases of a 100% crystalline sample of pure PVDF [66].

The porosity of the membranes was obtained by pycnometry by applying Equation (3):

(3) $$\varepsilon =\frac{m_{2}-m_{3}-m_{S}}{m_{1}-m_{3}}\times 100,$$

where m_S_ is the mass of the sample, m_1_ is the mass of the pycnometer filled with ethanol, m_2_ is the mass of the pycnometer filled with both ethanol and the sample, and m_3_ is the mass of the pycnometer with ethanol after removing the sample. The result is the average and standard deviation of three measurements for each sample.

The wettability of the samples was studied by contact angle measurements. The tests were carried out with a Dataphysics Contact Angle System OCA (San Jose, CA, USA) and 3 µL water drops deposited with a Hamilton SYR 500 µL 1750 N syringe (Giarmata, Romania). Six measurements were performed in each sample and the values are presented as the average and standard deviation.

Mechanical properties of the membranes were evaluated by stress–strain measurements using an AG-IS Shimadzu (Kyoto, Japan) with a 50 N load cell. Samples with 2.5 × 1 cm were stretched at a 1 mm/min rate until rupture.

## 3. Results and Discussion

The effect of the dissolution temperature and conditioning time before coagulation in the morphology of the membranes was studied by SEM. The top and bottom surfaces and cross section SEM images of the membranes are shown in Table 1 and Table 2, showing the effect of dissolution temperature and conditioning time, respectively.

Regarding the top surface, a wrinkled membrane with porous surface is obtained for the membrane 25 °C/0 min while the remaining membranes are smooth and with reduced surface porosity. In turn, the bottom surface is relatively smooth in all samples, with the exception of the samples 125 °C/0 min and 150 °C/0 min, which feature a larger roughness related to the higher temperature of the dope solution which typically leads to increased roughness in NIPS polymer processing [67]. It is also shown that all membranes present a heterogeneous porous cross section with finger-like pores, which is typical for PVDF-based polymers prepared by NIPS [64]. In fact, membrane morphology depends on the diffusion and transfer rates between solvent and non-solvent. Since DMF and water have high affinity, an instantaneous liquid–liquid separation occurs when the polymeric solution is immersed in the coagulation bath. Then, the non-solvent diffuses through the polymeric solution, first by the top surface in a very fast way, and then through the inner part of the membrane, resulting in an asymmetric transversal section with finger-like macropores on the top and regular micropores at the bottom [43]. Moreover, increasing polymer dissolution temperature leads to the formation of bigger regular micropores in the cross section, while it does not seem to significantly influence their distribution and homogeneity. In turn, the presence of the macropores remains in all samples, independent of the dissolution temperature [38]. In the cross-section SEM images of the membrane 125 °C/0 min finger like pores with shorter lengths compared to the other membranes can be observed, as well as with thicker pore walls.

The effect of the drying time on the morphology of the samples can also be evaluated by SEM images shown in Table 2. Regarding the samples dissolved at 25 °C, a slight difference in the top and bottom surfaces with increased drying time can be observed before immersion in the coagulation bath. In fact, increasing the drying time causes an apparent decrease of the pore size of the top surface, whereas for the bottom surface the opposite effect is observed: increasing pore size with increasing drying time. With regard to the cross section morphology of the membranes, there are no significant differences among them, the porosity and the pore distribution being similar for all membranes, and all feature an asymmetric transversal section with finger-like pores at the top and regular micropores at the bottom [43]. The size of the finger like pores tends, however, to be larger for higher drying times as can be observed in the samples 25 °C/5 min and 25 °C/10 min. Similar results are found for the remaining samples with different drying time, so the presented images are representative of the effect of drying time variation. Given the asymmetric morphologies obtained, rapid demixing occurred during phase inversion entering the unstable zone of the phase diagram [68].

**Figure 2:**
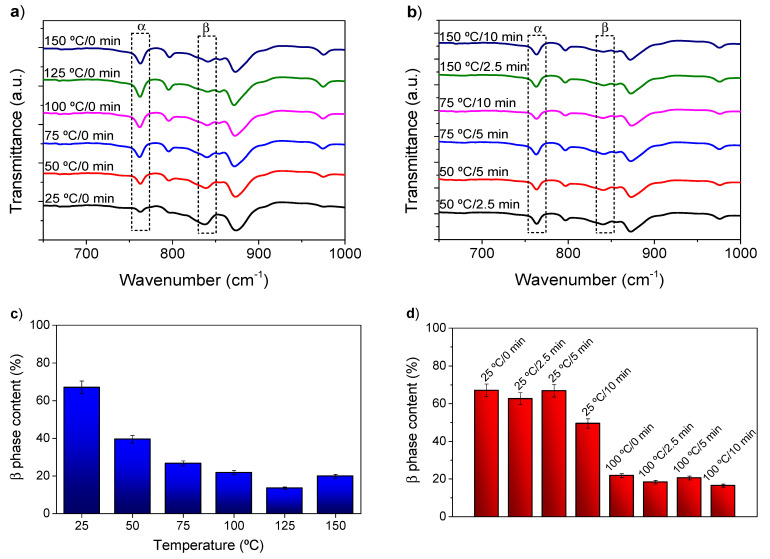
Representative Fourier transform infrared attenuated total reflection (FTIR-ATR) spectra of the different membranes as a function of: (**a**) dissolution temperature; (**b**) conditioning time and corresponding β phase content of the membranes as function of (**c**) dissolution temperature and (**d**) conditioning time.

Figure 2a,b shows representative FTIR-ATR spectra obtained to evaluate the influence of the dissolution temperature (Figure 2a) and drying time (Figure 2b) on the crystalline phases and β phase content (Figure 2c,d).

All samples show the peaks at 766 and 840 cm^−1^ corresponding to the α and β phases, respectively (Figure 2a,b), and no significant differences can be found between spectra. From the spectra of Figure 2a,b, the β PVDF content of the samples was determined using Equation (1), and the results show that the amount of β phase depends on the dissolution temperature (Figure 2c) but is not affected by the conditioning time before coagulation (Figure 2d). In fact, the β phase content tends to decrease with increasing polymer dissolution temperature. While a β phase content of ≈67% is obtained at a dissolution temperature of 25 °C, a value lower than 20% is obtained for temperatures higher than 100 °C. A slight increase in β phase concentration is noticeable for the samples produced at 150 °C when compared to those prepared at 100 °C, which is attributed to the decrease in the relative concentration of the polymer due to solvent evaporation, an effect previously reported for this PVDF/DMF system [69]. These results are attributed to the lower stability of the β phase at higher temperatures, thus with the α phase of the PVDF prevailing [70]. The decrease of the β phase is described in the literature, for PVDF/N-methyl-2-pyrrolidone systems, when the temperature of the coagulation water bath increased from 20 to 60 °C [51], which is attributed to the lower stability of the β phase at higher temperatures, compared to the α phase [71]. In turn, the conditioning time did not affect significantly the β phase content of the samples, with content variations within experimental error for the same dissolution temperature. Since the presence of the highly polar and electroactive β-PVDF is essential in a variety of applications, ranging from sensors and actuators to battery applications, the tailoring of its content through the dissolution temperature represents a useful and suitable approach.

**Figure 3:**
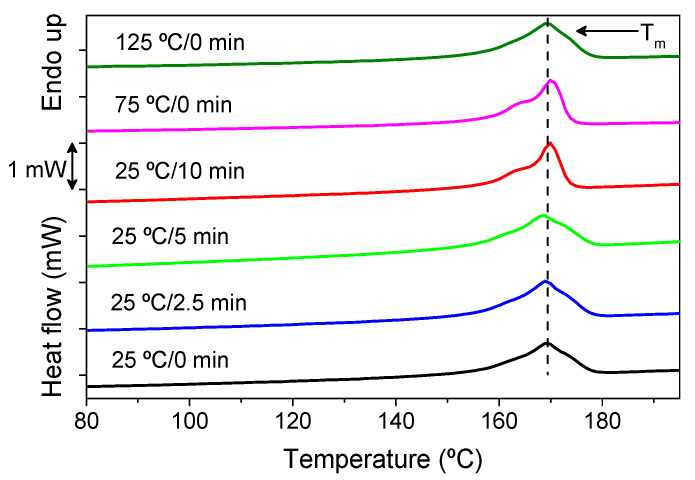
Differential scanning calorimetry (DSC) thermograms of representative PVDF membranes.

Regarding the influence of the NIPS processing parameters on the thermal properties and degree of crystallinity, DSC measurements were performed on representative PVDF membranes, as presented in Figure 3.

Figure 3 shows that the different processing conditions do not induce significant shifts of the peak corresponding to the PVDF melting temperature, occurring between 160 and 180 °C for all membranes, which is in agreement with the literature [72,73,74]. The melting enthalpy obtained from the area underneath the melting peak of each thermogram, and the degree of crystallinity calculated after Equation (2) are presented in Table 3 (uncertainty of 5%).

It can be concluded that the degree of crystallinity is similar for all membranes, with a mean value between 50% and 60%, which is typical for PVDF membranes [65]. The decrease of β phase content does not induces significant changes in the crystallinity. In fact, the increase in temperature of the medium, and the consequent increase in diffusion between solvent and non-solvent, does not significantly affect the degree of crystallinity [75].

Since the results demonstrate that the variations in the conditioning time before coagulation did not significantly affect the properties of the membranes, the following will just presented the results of the samples prepared with varying dissolution temperature.

**Figure 4:**
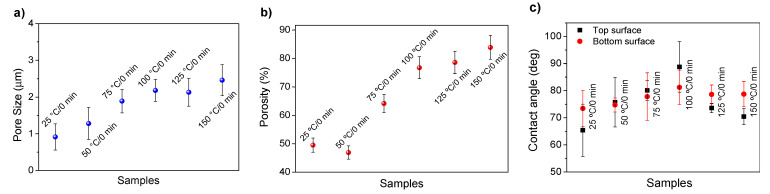
(**a**) Cross section pore size (excluding the finger like macropores), (**b**) porosity and (**c**) top and bottom surface water contact angles of representative PVDF membranes as a function of the dissolution temperature.

Figure 4a,b show the mean pore size of the cross section and porosity of representative PVDF membranes as a function of the dissolution temperature, respectively. Note that the finger like pores were not taken into account for the determination of the cross section mean pore size, since they are a well-known consequence of PVDF membranes production by NIPS [64]. The assays were not performed on the PVDF membranes as a function of the conditioning time before immersion on the coagulation bath since the SEM images reveal lower variation of the pore morphology and size when compared to the membranes prepared by dissolution temperature variation. Since wettability of the membranes is an important characteristic in many (bio)technological applications, including filtration membranes, surface contact angle measurements were performed in both surfaces of representative PVDF membranes as a function of the dissolution temperature; the results are shown in Figure 4c.

The dissolution temperature influences the pore size and porosity of the membranes, and there is a trend to larger pore formation with increasing dissolution temperature (Figure 4a). The formation of bigger pores can be directly related to the increasing dissolution temperature, since this leads to an increase in the solvent evaporation kinetics. The membrane with smaller pores is the 25 °C/0 min, showing mean pore size of ≈0.9 µm. Within the pores of this membrane there are smaller ones ranging from ≈0.3 to 0.5 µm, as shown in the cross-sectional SEM images of Table 1 and Table 2. This morphology has an important role on membrane behaviour, since the interconnectivity between the internal pores contributes to avoid membrane fouling that severally affects membrane performance [76]. The top and bottom surfaces did not present a quantity of pores that allows a precise establishment of a mean pore size, however, in the top surface the size of the pores present in the micrographs range between ≈0.3 and 0.7 µm, while for the bottom surfaces of the samples 125 °C/0 min and 150 °C/0 min the pore size ranges between ≈0.3 and 0.5 µm, respectively.

Regarding the porosity results (Figure 4b) a trend to a slight increase with increasing dissolution temperature can be found, from ≈50 to 83% for 50 °C/0 min and 150 °C/0 min membranes, respectively. A similar effect was observed in hollow-fiber PVDF membranes, which showed an increase of ≈10% in the porosity with increasing coagulation bath temperature up to 60 °C [52].

Contact angle values (Figure 4c) range between ≈60 and 90° on the top membrane surface and between ≈70 and 85° on the bottom one. Since the values of the contact angle are lower than 90° for all membranes spontaneous water intrusion to the pores will occur without the need of extra pressure [77]. Maximum contact angle values are obtained for the membrane 100 °C/0 min, both on the top and bottom surfaces, and the minimum values are obtained in the membrane 25 °C/0 min. These differences can be related to the number of pores present in the surfaces of the membranes, since the membranes 75 °C/0 min and 100 °C/0 min are those that present the smallest number of pores on both surfaces, as shown in the SEM images of Table 1, and in turn the remaining membranes present a larger number of pores on the top and bottom surfaces. The increase in the hydrophobicity of the membranes surface has been reported as an effect of the increase in the temperature of the medium, with temperatures of the coagulation bath up to 60 °C [51].

The measurement of mechanical properties of membranes is also a relevant parameter for (bio)technological applications, as they are related to the mechanical stability of the membrane. Thus, stress–strain measurements were performed on representative PVDF membranes. Molecular weight of the polymer, degree of crystallinity and microstructure of the membranes are properties that particularly influence the mechanical properties of PVDF-based membranes. Since the crystallinity remains approximately constant among samples, the variation of the mechanical properties will depend mostly on their morphology. Thus, the measurements were performed in PVDF membranes as a function of the dissolution temperature of a representative sample.

**Figure 5:**
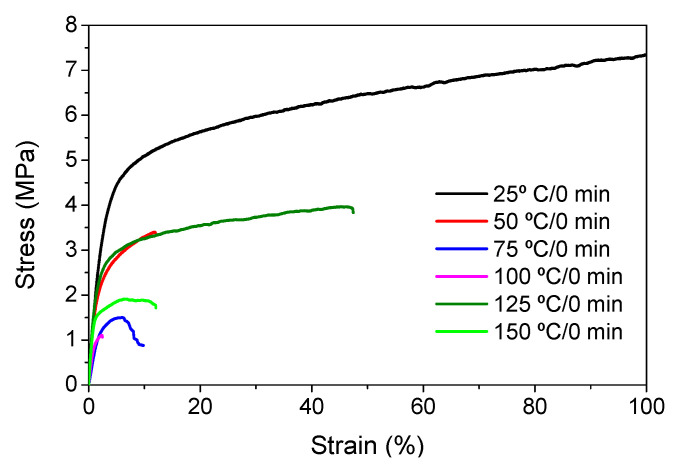
Stress–strain curves of representative PVDF membranes prepared at different dissolution temperatures.

Through these assays, it was possible to obtain the Young’s modulus, yield strain and strain at break of the membranes. These parameters are essential to determine the physical integrity of the membranes for applications. Figure 5 shows the mechanical stress–strain curves of the PVDF-based membranes.

The Young’s modulus (Table 4) was obtained for deformations at ≈1% in the elastic strain regime of the samples (uncertainty of 5%).

It is not possible to establish a direct correlation between the Young’s modulus and the dissolution temperature, however, a possible explanation for the higher values obtained for samples 25 °C/0 min, 50 °C/min and 125 °C/0 min might be related to the cross-sectional morphology of the membranes. The membranes 25 °C/0 min and 125 °C/0 min present lower macro void finger-like pores at the top of the cross section (Table 1), and the membranes 25 °C/0 min and 125 °C/0 min show lower porosity (Figure 4b). These features confer on these membranes a higher Young’s modulus and strain at break. In addition, the walls of the pore cavities of the membrane 125 °C/0 min are thicker than the others, which leads to the higher Young’s modulus obtained for these membranes (Table 1). These values range from 70 to 300 MPa for 75 and 25 °C, respectively, and should be noted that the values are in agreement with the values found in the literature for similar systems [78]. The yield strain is similar for all membranes, ranging from 1.0% to 1.8%. However, the strain at break is higher for the membrane 25 °C/0 min, which can be attributed to the simultaneous lower pore size and porosity. For smaller pores, the membrane is harder to break, and so the breaking occurs at ≈81% of the elongation.

## 4. Conclusions

PVDF-based membranes were produced by non-solvent induced phase separation (NIPS) with DMF as solvent and water as non-solvent. The polymer dissolution temperature and the conditioning time before immersion in the coagulation bath were varied and their effect on the morphological, structural, thermal and mechanical properties of the PVDF membranes was evaluated. Membranes with different morphologies were obtained, with distinctive finger-like pores and a porous cross section structure that allows transversal flow along the membrane. Pore size values between ≈0.9 and 2.1 µm were obtained for the membranes prepared by polymer dissolution at 25 °C and 125 °C, respectively. A gradual increase in pore size with dissolution temperature was verified. Porosity also increases gradually with increasing dissolution temperature, ranging from ≈50 to 90% for membranes prepared at 50 °C and 150 °C, respectively. The water contact angle varies between 59 and 80° for the top surface (subject to air drying) and between 73 and 81° for the bottom surface (in contact with the glass). The polymer polar and electroactive β phase decreases with increasing dissolution temperature and the degree of crystallinity ranges from 52% to 59% for the different membranes. It is not possible to correlate the processing parameters with the overall mechanical properties, nevertheless these values follow the same trend of the crystallinity variations. Thus, it is concluded that the control of the dissolution temperature and conditioning time before coagulation during NIPS processing represents a suitable way to tailor the bulk and surface (top and bottom) properties of PVDF polymer membranes, namely surface morphology, porosity and pore size, in order to meet specific application requirements.

## Figures and Tables

**Table 1:** Representative scanning electron microscopy (SEM) images of the top and bottom surfaces and cross section of the poly(vinylidene fluoride) (PVDF) membranes for different dissolution temperatures.

Effect of Varying Dissolution Temperature				
	Top Surface	Bottom Surface	Cross Section	
25 °C/0 min	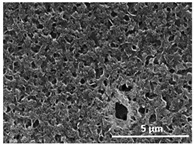	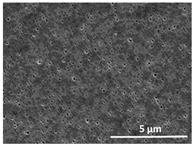	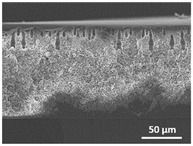	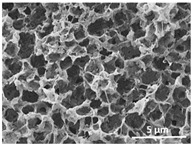
50 °C/0 min	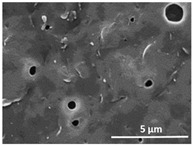	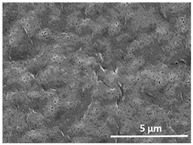	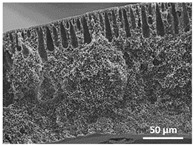	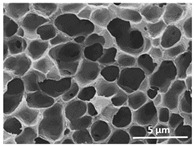
75 °C/0 min	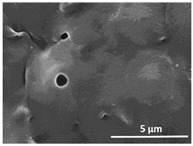	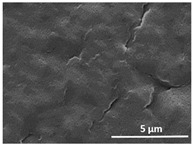	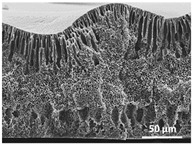	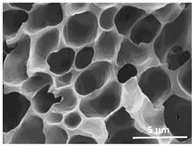
100 °C/0 min	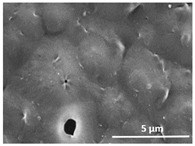	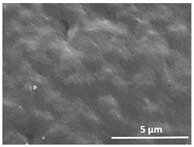	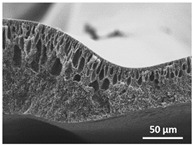	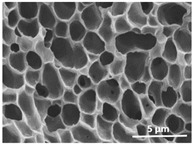
125 °C/0 min	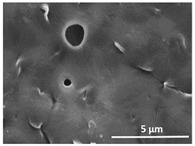	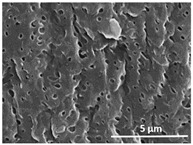	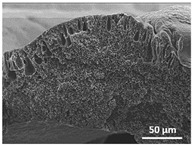	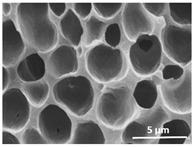
150 °C/0 min	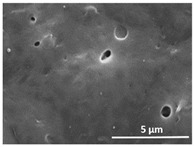	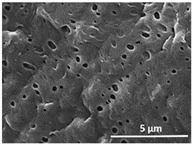	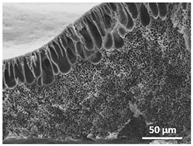	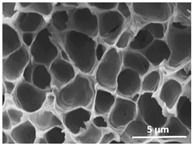

**Table 2:** Representative SEM images of the top and bottom surfaces and cross section of the PVDF membranes for different conditioning time.

Effect of Varying Drying Time				
	Top Surface	Bottom Surface	Cross Section	
25 °C/0 min	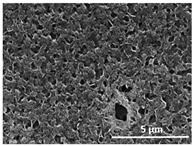	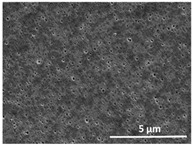	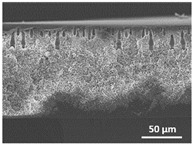	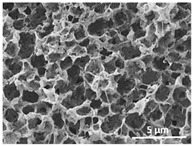
25 °C/2.5 min	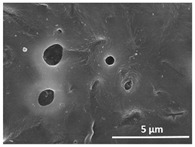	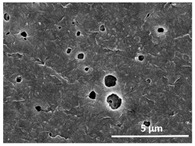	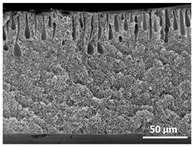	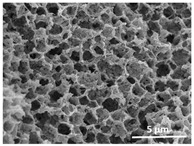
25 °C/5 min	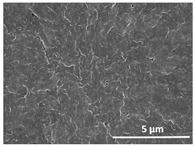	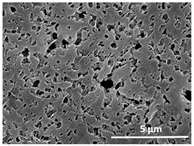	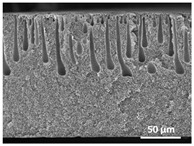	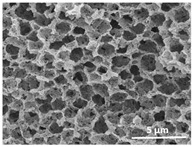
25 °C/10 min	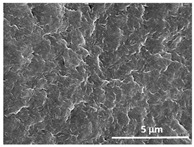	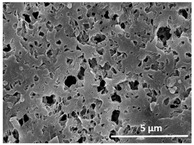	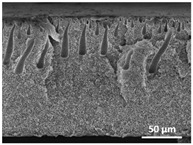	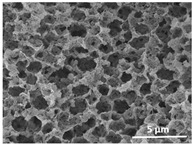

**Table 3:** Melting enthalpy and degree of crystallinity of different PVDF membranes.

Sample	25 °C/0 min	25 °C/2.5 min	25 °C/5 min	25 °C/10 min	75 °C/0 min	125 °C/0 min
ΔH_f_ (J/g)	57.2 ± 2.9	50 ± 2.5	58.7 ± 2.9	54.5 ± 2.7	50.1 ± 2.5	52.2 ± 2.6
χ_c_ (%)	57.5 ± 2.9	50.6 ± 2.5	59.2 ± 3.0	55.7 ± 2.8	52.2 ± 2.6	55.1 ± 2.8

**Table 4:** Young’s modulus, yielding strain and strain at break of representative PVDF membranes as a function of dissolution temperature.

Sample	Young’s Modulus (MPa)	Yield Strain (%)	Strain at Break (%)
25 °C/0 min	300 ± 15	1.6 ± 0.1	81.2 ± 4.1
50 °C/0 min	200 ± 10	1.4 ± 0.1	12.0 ± 0.8
75 °C/0 min	70 ± 4	1.8 ± 0.1	6.4 ± 0.3
100 °C/0 min	100 ± 5	1.0 ± 0.2	2.5 ± 0.3
125 °C/0 min	200 ± 13	1.9 ± 0.1	47.4 ± 3.1
150 °C/0 min	100 ± 6	1.1 ± 0.3	12.0 ± 2.0

## Data Availability

The data presented in this study are available on request from the corresponding author.
